# Navigating inequities in the delivery of youth mental health care during the COVID-19 pandemic: perspectives of youth, families, and service providers

**DOI:** 10.17269/s41997-022-00670-4

**Published:** 2022-07-19

**Authors:** Toula Kourgiantakis, Roula Markoulakis, Amina Hussain, Eunjung Lee, Rachelle Ashcroft, Charmaine Williams, Carrie Lau, Abby L. Goldstein, Sugy Kodeeswaran, Anthony Levitt

**Affiliations:** 1 Factor-Inwentash Faculty of Social Work, University of Toronto, 246 Bloor Street West, Toronto, ON M5S 1V4 Canada; 2 Family Navigation Project, Sunnybrook Research Institute, Toronto, ON Canada; 3 Department of Occupational Science and Occupational Therapy, University of Toronto, Toronto, ON Canada; 4 Department of Applied Psychology and Human Development, Ontario Institute for Studies in Education, University of Toronto, Toronto, ON Canada; 5 Family Navigation Project, Sunnybrook Health Sciences Centre, Toronto, ON Canada; 6 Faculty of Medicine, University of Toronto, Toronto, ON Canada

**Keywords:** Youth, Families, Mental health, Navigation, COVID-19 pandemic, Equity, Jeunes, familles, santé mentale, intervention-pivot, pandémie de COVID-19, équité

## Abstract

**Objectives:**

There have been concerns about the adverse effects of the COVID-19 pandemic on Canadian youth (aged 16–24) as they have the highest rates of mental health concerns. The objectives of the present study were to explore the experiences of youth with mental health and/or addiction concerns and their families during the pandemic, and to examine how adequate and equitable mental health services have been for youth and families from the perspectives of youth, parents, and service providers.

**Methods:**

Using a descriptive qualitative research design and a university–community partnership, we conducted individual interviews with youth, parents, and service providers. The study involved a total of 25 participants (*n*=15 service users, *n*=10 service providers). Among the service users, 11 participants were parents and four were youth. We used thematic analysis to analyze interview data.

**Results:**

The thematic analysis identified three themes in the data: (1) youth mental health concerns have increased, whereas supports have decreased, (2) families end up being the treatment team with increased burden, little support, and lack of recognition, and (3) inadequate and inequitable mental health services for youth and families are amplified during the pandemic.

**Conclusion:**

At a time when mental health needs were higher, the mental health care system offered less support to youth and their families. For a more equitable response to the pandemic, we need an accessible and integrated mental health care system that shows a commitment to addressing social determinants and reducing health disparities and inequities in access to mental health services.

## Résumé

**Objectifs:**

Les effets indésirables de la pandémie de COVID-19 sur les jeunes (16 à 24 ans) du Canada suscitent des inquiétudes, car ce sont les jeunes qui présentent les taux les plus élevés de problèmes de santé mentale. Nous avons voulu explorer les expériences de jeunes aux prises avec des problèmes de santé mentale et/ou de toxicomanie et de leurs familles durant la pandémie, et à déterminer si les services de santé mentale sont suffisants et équitables pour les jeunes et leurs familles du point de vue de jeunes, de parents et de prestataires de services.

**Méthode:**

À l’aide d’un plan de recherche qualitative descriptive et d’un partenariat entre les milieux universitaire et associatif, nous avons mené des entretiens individuels avec des jeunes, des parents et des prestataires de services. Vingt-cinq personnes ont participé à l’étude (*n* = 15 utilisateurs et utilisatrices de services, *n* = 10 prestataires de services). Parmi les utilisateurs et utilisatrices de services, 11 étaient des parents et 4 étaient des jeunes. Nous avons eu recours à l’analyse thématique pour analyser les données des entretiens.

**Résultats:**

Trois thèmes se sont dégagés de l’analyse thématique des données : 1) les problèmes de santé mentale des jeunes augmentent, tandis que les mesures d’aide diminuent, 2) ce sont les familles qui finissent par constituer l’équipe de traitement, ce qui représente un fardeau accru, peu de soutien et un manque de reconnaissance, et 3) l’insuffisance et l’iniquité des services de santé mentale offerts aux jeunes et à leurs familles ont été amplifiées durant la pandémie.

**Conclusion:**

Alors que les besoins en santé mentale étaient plus élevés, le système de soins de santé mentale a offert moins de soutien aux jeunes et à leurs familles. Pour une intervention plus équitable face à la pandémie, nous avons besoin d’un système de soins de santé mentale accessible et intégré, manifestement engagé à aborder les déterminants sociaux et à réduire les disparités d’état de santé et les iniquités d’accès aux services de santé mentale.

## Introduction

The COVID-19 pandemic has had a significant negative impact on mental health worldwide (Fegert et al., 2020). In Canada, there have been concerns about the adverse effects on youth (aged 16–24) as this age group has the highest rates of mental health concerns (20%), the highest rates of substance use disorders (11.9%), and the most unmet mental health care needs even pre-pandemic (Sukhera et al., 2017). Youth aged 16–24 are in a developmental transition between childhood and adulthood and the stage in which most mental illnesses have their onset (MHCC, 2015). While most mental health concerns emerge during this developmental period, there are multiple access barriers for youth seeking mental health care, such as fragmented services, long waitlists (Sukhera et al., 2017), geographical challenges, high costs, stigma, racism, discrimination, and lack of culturally responsive treatment and care (Fante-Coleman & Jackson-Best, 2020; MHCC, 2015). Access is defined as a multidimensional concept which includes “access to a service, a provider or an institution…[and] the opportunity or ease with which consumers or communities are able to use appropriate services in proportion to their needs” (Levesque et al., 2013, p. 1). For youth who are part of equity-deserving groups, there are even greater disparities to mental health care access, and they face systemic and structural barriers related to racism, stigma, and discrimination (Craig et al., 2021; Fante-Coleman & Jackson-Best, 2020). According to the World Health Organization (WHO, 2021), health equity refers to a commitment to reduce or eliminate avoidable or unfair outcomes, disparities, and marginalization faced by some groups or populations. There is a need for services to better engage youth and their families because 52% of youth drop out of mental health treatment (Singh et al., 2010). Although parents or caregivers play an important role helping youth access appropriate treatment and supporting youth in their recovery (Markoulakis et al., 2020), the lack of services and inadequate involvement of families in treatment creates an enormous burden on families (Miller et al., 2017). Service providers have also reported “feeling lost” in how to treat youth in the fragmented mental health care system in Canada (Miller et al., 2020).

The COVID-19 pandemic has had emergency measures such as lockdowns, social distancing, and school and employment disruptions that have amplified mental health concerns in youth (Fegert et al., 2020; Jones et al., 2021). Since the outbreak of the pandemic, youth have experienced a decline in their mental health (Cost et al., 2021) and an increase in substance use (Bartel et al., 2020; Statistics Canada, 2020). Families have also experienced increased stress related to school and childcare closures, employment loss and changes, financial insecurity, and deteriorating mental health in children (Gadermann et al., 2021). There is growing evidence of disproportionate impacts of the pandemic on mental health linked to social determinants of health such as employment and income, gender, sexual orientation, racism, housing, disability, and social supports (Jenkins et al., 2021). Studies show higher rates of mental health concerns since the start of the pandemic among sexual and gender minority youth (Hawke et al., 2021a; Jones et al., 2021), youth with disabilities (Arim et al., 2020), and Black, Indigenous, and People of Colour (BIPOC) populations (Castro-Ramirez et al., 2021). There has also been reduced access to services (Fegert et al., 2020; Hawke et al., 2020) and widened inequities in the delivery of mental health care (Ashcroft et al., 2021) since the start of the pandemic. The pandemic has exposed and exacerbated mental health inequities in youth defined by the WHO as “avoidable and unfair differences in health status between groups of people or communities” (WHO, 2021).

Research to date has largely focused on quantifying the impacts of COVID-19 on the mental health and service access challenges faced by youth and their families (Bartel et al., 2020; Gadermann et al., 2021; Hawke et al., 2020; Jenkins et al., 2021), but few studies have attended to the *lived experiences* of youth with mental health and addiction concerns and their caregivers during COVID-19. Qualitative research provides a deeper understanding of the impact of COVID-19 on youth and their families, and it can provide insights to inform policies to address the growing mental health care needs of this population. This is best accomplished through the involvement of multiple informants, each providing unique and complementary perspectives on youth mental health and service needs. Engaging youth and their families in research ensures that adjustments to services and pandemic responses are developmentally appropriate and culturally responsive to reflect the needs identified by those who are most affected (MHCC, 2015). Furthermore, engaging service providers in research can provide insight on their perspectives of youth mental health care and system capacities for addressing them (Miller et al., 2020). To address some of these gaps, the present study explored the impact of COVID-19 on youth and families from the perspectives of youth, parents/caregivers, and service providers. These findings are part of a larger study examining facilitators and barriers for families seeking mental health services for youth. The following research questions guided this study: What are the experiences of youth with mental health and/or addiction-related concerns and their families during the COVID-19 pandemic? How adequate and equitable have services been in response to COVID-19 for youth and families coping with mental health and addiction-related concerns?

## Methods

### Design and setting

A descriptive qualitative research design guided this study, and this is a suitable approach for a topic that has received limited empirical exploration (Sandelowski, 2010). We also used a collaborative research approach that engages knowledge users and permits researchers and community partners to co-produce knowledge (Jull et al., 2019). This approach supports an inclusive and recovery-oriented framework in which the lived experiences of service users are recognized as an integral component for improving systems of care (Jull et al., 2019; Piat & Sabetti, 2009). This study was conducted through a collaborative partnership between the University of Toronto and the Family Navigation Project (FNP) at Sunnybrook Health Sciences Centre. We also had an advisory committee consisting of a youth, a parent, and service providers. The Family Navigation Project was founded “by families, for families” and offers navigation services to families and youth, connecting them to appropriate mental health and addiction services. FNP is connected to more than 1100 service providers and programs across Ontario and is the largest navigation program in Canada, serving approximately 750 families annually (Markoulakis et al., 2016, 2019). Ethics approval was received through the research ethics boards at the University of Toronto and Sunnybrook Health Sciences Centre.

### Participant sample and recruitment

A purposeful sampling strategy (Patton, 2002) was used to obtain rich and diverse perspectives from service users and service providers with lived experience and expertise in youth and family mental health. Recruitment was conducted in collaboration with FNP. Service providers were eligible to participate if they had received a referral for youth and/or parents/caregivers between January 2019 and July 2021. This included service providers working in hospitals, community-based settings, and private practice. Navigators at FNP were also eligible to participate as they worked directly with families and youth served by the program. Service users who were eligible included youth (aged 16–24) who received navigation support for mental health and/or addiction-related services and parents/caregivers who received services from the FNP. Participants were given a $25 gift card as an honorarium for their time.

### Data collection

Most of the interviews were conducted by the Research Coordinator (AH), who was supervised by the Principal Investigator and first author (TK). Data collection occurred from March 2021 to July 2021 which coincided with the third wave of COVID-19 in Ontario. In adherence with physical distancing measures, all participant interviews were conducted via telephone or online using a video conferencing platform and they were 60 to 90 minutes in length.

We used a semi-structured interviewing format (DiCicco-Bloom & Crabtree, 2006) with an interview guide consisting of open-ended questions that were grouped under the following categories: (1) experiences of youth, parents/caregivers, and service providers; (2) previous and/or current mental health services; (3) presenting mental health concerns for accessing services; (4) facilitators and barriers to accessing mental health and addiction services; (5) role of families in services, healing, and recovery; and (6) experiences of stigma, racism, and discrimination. Each category had a question that asked participants to describe how this was affected by the current context with COVID-19. Participants completed a demographic questionnaire online and all interviews were audio-recorded and transcribed with participants’ consent. Each participant was assigned an ID code to preserve anonymity. Only the Research Coordinator (hereafter, RC) and Principal Investigator (hereafter, PI) had access to the raw data.

### Data analysis

To analyze our data, we used thematic analysis, a method for identifying themes and patterns within data (Braun & Clarke, 2006). Thematic analysis is a six-stage process that includes (1) data familiarization; (2) generating initial codes; (3) generating initial themes from coded data; (4) reviewing themes; (5) defining and developing names of themes; and (6) interpreting and reporting (Braun & Clarke, 2006). We used the online software Dedoose to organize and synthesize data. Data analysis was completed by the PI, RC, and two research assistants (RAs). In the first phase of analysis, the four research team members familiarized themselves with the data by reading the transcripts and writing memos. We created a codebook that provided detailed descriptions of the codes with exemplars. In the second phase, these four team members identified initial codes that emerged from their review. This was a recursive process that involved constant movement between generating and defining codes, reading through transcripts, and adding and refining codes as needed (Braun & Clarke, 2006). The RAs were assigned transcripts as first or second coders. All transcripts were coded by the first coder and reviewed by the second coder, and the PI or RC reviewed each double-coded transcript to resolve discrepancies and ensure there was consensus. Discrepancies were debriefed in our weekly meetings to help us arrive at consensus and update our codebook when needed. When coding was completed, we reviewed the codes with their excerpts and identified overarching themes. We cross-checked the themes with other research team members, including our community partner and advisory committee.

Rigour and trustworthiness in this study were enhanced through credibility, dependability, confirmability, and transferability (Lincoln & Guba, 1985). Theme development was a reflexive approach for research team members. We recognize that our diverse identities, experiences, and assumptions can influence coding and theme development. Our research team consists of researchers, service providers, peers, and students with diverse identities including but not limited to race, ethnicity, religion, gender, ability, and age. We also had research team members with many years of clinical experience in community mental health, as well as members with lived experiences. To make our bias and lived experience transparent and have our data interpretation stay closely aligned with participants’ words, the research team wrote reflexive memos during analysis, debriefed regularly throughout the coding process, and kept thorough notes of all meetings. Other strategies used to enhance trustworthiness included triangulation of data sources, prolonged engagement with data, use of field notes during data collection, thick description of participant quotes, and researcher triangulation.

## Results

The study involved a total of 25 participants (*n*=15 service users, *n*=10 service providers). Among the service users, 11 participants were parents and four were youth. Most of the parents were mothers (*n*=10) with one father participant. Eight parents were supporting a son and three were supporting a daughter with mental health and/or addiction concerns. These sons/daughters were not part of the study. Table 1 provides more information about participant characteristics.

**Table 1:** Participant characteristics

Characteristics, *n* (%)	Parents/caregivers (*n*=11)	Youth (*n*=4)	Service providers (*n*=10)
Gender			
Male	1 (9%)	-	1 (10%)
Female	10 (91%)	4 (100%)	8 (80%)
Did not identify	-	-	1 (10%)
Age (mean)	57	21	-
Self-identified ethnoracial identity			
Black	-	1 (25%)	-
South Asian	1 (9%)	1 (25%)	1 (10%)
East Asian	2 (18%)	1 (25%)	-
White	7 (64%)	1 (25%)	8 (80%)
Mixed race/biracial	1 (9%)	-	1 (10%)
Parent role			
Mother	10 (91%)	-	-
Father	1 (9%)	-	-
Youth supported by parents/caregivers			
Son	8 (73%)	-	-
Daughter	3 (27%)	-	-
Highest level of education			
High school	-	3 (75%)	-
College diploma/undergraduate university	8 (73%)	1 (25%)	-
Graduate degree university	3 (27%)	-	10 (100%)
Educational degree			
Master of social work	-	-	8 (80%)
Master of education	-	-	2 (20%)
Identified role for service providers			
Social worker	-	-	1 (10%)
Crisis worker	-	-	1 (10%)
Clinician navigator	-	-	7 (70%)
Did not identify	-	-	1 (10%)
Practice setting			
Hospital	-	-	6 (60%)
Both hospital and private practice	-	-	3 (30%)
Post-secondary institution	-	-	1 (10%)
Mean years of practice experience	-	-	10.6
Type of concerns^a^			
Mental health	4 (36%)	3 (75%)	-
Substance use/behavioural addictions	1 (9%)	-	-
Concurrent disorders	6 (55%)	1 (25%)	-

^a^Type of concerns for the parent/caregiver group refers to the self-reported mental health and/or addiction concerns of the youth they are supporting

Our thematic analysis identified three themes in the data: (1) youth mental health concerns have increased, whereas supports have decreased; (2) families end up being the treatment team with increased burden, little support, and lack of recognition; and (3) inadequate and inequitable mental health services for youth and families are amplified during the pandemic.

### Youth mental health concerns have increased, whereas supports have decreased

Participants described increased mental health and/or addiction concerns in youth during the pandemic. One service provider highlighted, “We’ve seen a huge spike in the ER of adolescents…they’re socially isolated” (P12, SP). Many participants underlined that anxiety and depression had increased. One service provider stated, “Anxiety has dramatically increased. Same with depression” (P8, SP). Although mental health concerns in youth pre-dated the pandemic, these concerns were amplified during the pandemic. One parent stated, “The pandemic has affected her very much…she’s freaking out on cleaning…I would have to stand outside and wipe everything coming and wipe my shoes and oh, my, my, that was very difficult to live through with her” (P11, SU-P). Some participants spoke about safety concerns for youth during the pandemic. According to one service provider, “There might have been some underlying mental health issues pre-pandemic, but it’s really been exacerbated to the point of crisis…families are concerned around suicide for their loved ones” (P2, SP). Another service provider stated, “There’s been a bit of an increase in terms of disordered eating referrals” (P25, SP), and another added, “I know that I’ve seen more OCD than I’ve seen in any of my time here” (P1, SP).

Lockdown measures during the pandemic created additional increased stressors and social isolation for youth which further aggravated mental health and addiction concerns. A youth expressed, “You’re trapped up, there’s nothing outside, like you know, what it is to be a drug addict, and to be depressed, and then the whole city’s locked down? The only thing you have to do is to stay in your house and to do drugs” (P23, SU-Y). One service provider observed that “staying home means disruptions in sleep, routine, hygiene, appetite, and lack of socialization.” (P17, SP).

Service providers and parents discussed concerns and uncertainty related to excessive technology use in youth during the pandemic. One service provider explained,

I have a lot of families that their youth is engaging more than ever in technology and substances and the family members are unsure if this is normal behaviour in response to the pandemic or if it’s problematic... some of them are taking it away because that’s what they might have done pre-pandemic, and then they’re seeing an escalation in their youth’s mental health or suicidality. (P2, SP)

Many participants stressed that not having in-person school was negatively impacting youth mental health due to “a loss of support” (P19, SP). A service provider stated, “failing courses, unable to participate effectively in school…problems accessing help from the teacher or from the school…can lead to issues with mental health” (P17, SP). Another service provider explained, “if you’re living within a family that has different beliefs…about mental health, and as a youth, you’re not able to go to school, or have those places outside of your family…there’s the barrier” (P19, SP).

Others also reported abrupt changes to services during the pandemic, with some services no longer being available and others being offered virtually. One youth underlined, “I feel like during COVID, it’s just been extra difficult to get proper help” (P21, SU-Y). Many service providers highlighted the negative effects of not having in-person support services to focus on behavioural activation, coping, and skill development. One service provider explained,

The type of support they’re able to get now through virtual is just not the same…people aren’t able to practice the skills. Regular kind of behaviour activation isn’t happening…They’re regressing and getting frustrated…A whole part of somebody’s life was kind of shut down, and you can cope for a certain amount of time, and then things get harder. (P19, SP)

A parent described that their youth had recently accessed mental health and addiction treatment at a hospital, yet once the pandemic was declared, “everything was shut down, and it was going to take too long” (P5, SU-P). Challenges finding services during the pandemic were also described by a youth who stated, “walk-in places that I thought would have been open are closed, especially during lockdown, everything is actually really closed. I really felt like I couldn’t get help…It made me feel hopeless. My last resort was to call a suicide line” (P15, SU-Y). Another youth expressed, “I’ve talked to lots of places but lots of places have waiting lists. I have addiction and depression! I don’t know how people can put you on a waiting list” (P23, SU-Y). A parent stated, “His psychiatrist hasn’t seen him physically since COVID…When you have mental health issues…he needs to be in front of a person” (P22, SU-P). A youth emphasized the benefit of having the support of a navigator while other services were unavailable: “I’m filled with gratefulness. If I didn’t have her then I wouldn’t really know where I would be right now” (P15, SU-Y).

Participants described a lack of specialized services to address specific mental health concerns or neurodevelopmental needs. A youth stated: “A lot of the groups that were referred to me…hardly any of them were OCD-specific, and that was really frustrating” (P21, SU-Y). A few service providers noted the decrease in services for youth with autism. One service provider underlined, “They’re not able to access the programs that were really able to provide routine and momentum in terms of skill building” (P14, SP). Another service provider identified gaps in services for LGBT youth during the pandemic: “The services are already so limited, especially in the public sector! And then even private…sometimes to find a private clinician who specializes in the LGBT population can be very hard” (P1, SP). Participants also described a lack of services for substance use disorders for youth, including a gap in residential treatment. One parent explained that her son was “snorting fentanyl, 15, 17 times a day” and noted, “the government is just hopeless…we don’t really have consumption sites for youth, we don’t have residential treatment for youth…Our status quo is we’re letting youth, no matter how young, play Russian roulette with their lives” (P10, SU-P). Another parent expressed concerns related to the lack of services: “I’m looking at the effects of COVID and thinking, this is a tsunami coming. This is a mental health disaster on our hands. Kids are literally dying because they can’t get those services” (P9, SU-P).

### Families end up being the treatment team with increased burden, little support, and lack of recognition

Most participants emphasized the important role of families in supporting youth with mental health and addiction concerns and the challenges for caregivers who had assumed additional responsibilities since the start of the pandemic:

Families are experiencing COVID exhaustion…families are struggling because they’re having to work from home…And having to manage that while managing what’s happening with their kids…while living in a pandemic. And not having access to other people, like grandparents, or community…to sort of help out, give them a break…Families are essentially becoming the treatment team for their youth. (P14, SP)

A youth expressed hesitation to discuss her concerns with her family as they have other concerns and responsibilities. “My family doesn’t have time [my sister] has her kid, and she’s just 23, and she has her own addiction. I think my mom is emotionally exhausted…and she just doesn’t have it in her anymore” (P23, SU-Y). A service provider stated that families have additional burden because “there’s probably nothing out there” in terms of supports and services during the pandemic and they are feeling “fear, desperation, and hopelessness” (P14, SP).

Parents also described receiving mixed messages about how to support their youth during the pandemic. They continued to experience exclusion from their youth’s mental health care as they had pre-pandemic, but they also felt they needed to compensate for the unavailable or inadequate services. A parent described an incident in which she took her son to the emergency department because he was suicidal:

Being COVID I cannot accompany him, so I dropped him off…He went into emerg, and got seen by a doctor for, like, a whole two minutes. Got told that a social worker would come and see him…and he’s sitting out there waiting, texting me…‘nobody’s come to see me’... So, three hours later I say to him, ‘go up to the nursing station and ask them what’s going on’…And he’s getting agitated because he doesn’t have his mom with him… he goes…to the nursing station, to ask what’s going on, and they say, ‘oh, you’re waiting for a social worker, go back and sit down’…An hour later, the doctor comes back and says to him, ‘oh, our social worker left at 11:00’…They discharged him!…This is an individual that’s told you, ‘I want to kill myself’…I found my son walking on the road. (P22, SU-P)

Many participants discussed health care consent laws, confidentiality policies, organizational policies, and service provider practices that contribute to the exclusion of families from their youth’s mental health care. While these issues are not unique to the pandemic, they appeared to be exacerbated for parents who were coping with declining mental health in youth and fewer services. A parent stated that “age is a barrier” when calling a crisis helpline during the pandemic: “I was calling because he was very suicidal, and I needed help…They said…how old is your son, and I said, 19, and they said…I’m sorry, we can’t speak to you” (P22, SU-P). Another parent spoke about parents’ rights and responsibilities: “You have no rights as parents. You’re taking them to school, you are feeding them, you are lodging them, and you have absolutely no rights when it comes to mental health” (P11, SU-P). Some service providers noted that they continued dealing with challenges related to family involvement in youth mental health care: “It’s a balance between really honouring family-centred practice as well as honouring youth autonomy, and youth engagement” (P14, SP). A parent stated, “They didn’t care when he became homeless, no one was willing/able to help because as far as they were concerned, that was his right to refuse treatment” (P10, SU-P). Another parent noted that they were “not allowed” to be involved in their youth’s treatment “because she is an adult” but the support from a navigator helped reduce the burden: “It might not be too dramatic to say that the family navigator, with whom I was speaking, saved my life” (P9, SU-P).

Noteworthy is that all youth participants stated that they would have liked for service providers to connect with their families to help them better understand mental health and their youths’ experiences. One youth explained,

During the pandemic, it’s been extra difficult to get proper help…Every time I would go to receive help, [it would be helpful if] they would always offer support for my parents as well…Like, my culture, we don’t really take mental seriously, so I guess that’s the reason my parents don’t really have an understanding (P21, SU-Y).

### Inadequate and inequitable mental health services for youth and families are amplified during the pandemic

Participants described health disparities related to economic, sociocultural, and mental health system barriers. Many participants underlined difficulties accessing publicly funded mental health services due to long waitlists or services that had closed during the pandemic. According to one service provider, “we’re seeing waitlists of months… The private sector has always been known to be a lot faster, and we’re seeing a lot more of a wait in that area too” (P14, SP). With the lack of available and accessible public services, many participants mentioned the need for sufficient economic resources to pay for private mental health care. A youth stated, “I have to have money to be healthy” (P23, SU-Y). A parent linked cost with quality of care: “If you do not have a lot of money…then you do not have the opportunity to get the best quality treatment” (P20, SU-P).

While all participants recognized the benefits of navigation services, many participants expressed dissatisfaction with the quality of mental health services during the pandemic. One parent noted that the “family navigation people were the only ones to call back. The rest of them didn’t reply, which is inexcusable” (P9, SU-P). Another parent stated that service providers “really need to understand from the patient’s and the family’s perspective, and treat them as a person, not just another patient” (P24, SP). A youth explained that many services needed a referral from her doctor, but this created additional barriers: “[my doctor] wouldn’t ever reach out or answer the phone calls. So, it was really difficult to get connected with any other services” (P21, SU-Y). Several service providers described feeling overwhelmed by the increased demand since COVID, which impacted their service delivery. A service provider stated, “they don’t feel…they have the time, or the energy, especially during COVID…to really provide the service that the person requires” (P12, SP). Service providers emphasized an increase in workload since the start of the pandemic: “We are overloaded…there’s a really long waitlist. We’re really busy, this is the busiest I’ve ever been” (P18, SP). Another service provider noted, “we’re wanting to help so many people that sometimes we’re rushing to the outcome versus really being client-centred” (P14, SP).

Some participants described experiences of stigma, racism, and discrimination by mental health services and service providers and while these experiences were not caused by the pandemic, they amplified the difficult experiences for youth and families and increased barriers to mental health services. One youth described the treatment she received in a mental health care setting while accompanied by her mother: “They were, like, treating me and my mom as if we can’t understand a single word they’re saying. Or if I repeat three times that my mom can speak perfect English, they kind of would just be in disbelief” (P21, SU-Y). A Black-identified youth spoke about her parents’ reluctance to seek professional help and stated that for her parents “racism is a key barrier” to mental health service access (P13, SU-Y). A service provider expressed that families feel judged and marginalized: “For parents, there has been a history of feeling discrimination, of not developing a trusting relationship with the health care system, but developing one where they feel blamed, excluded and not represented” (P14, SP).

Among the mental health services that continued operating during the pandemic, most offered virtual rather than in-person services and for many participants this service delivery method was inadequate. A youth described barriers faced by her parents: “They don’t have money to turn on their internet…they don’t have internet to turn on their phone, how are they even supposed to call anybody to set up a meeting when they don’t even have a phone?” (P23 SU-Y). A service provider noted, “Virtual means of service is not viable…families are feeling like services are inaccessible. Few services are willing to do face to face…and they’re mostly *not* in the public sector” (P14, SP). Many service providers discussed virtual services not being a preferred treatment for youth. “I’ve heard from a lot of young people, that they want to do things in person. Youth are then delaying treatment because they’re waiting for things to re-open in person” (P1, SP).

Participants underlined that for youth with developmental disabilities, as well as some mental illnesses and addictions, the virtual format was inaccessible. A parent explained, “It’s not accessible for my son, who refuses to use those services…it’s just another barrier” (P22, SU-P). Participants explained that it is important for the service provider to see the youth to be able to effectively engage and assess. One parent stated, “it’s easy for him to say, I have no camera, which then I find is easy to hide…there’s something that can be said about seeing somebody physically and how they are, in terms of their demeanour” (P6, SU-P). Another parent described changes to the psychiatric services: “My daughter finds that the way that the psychiatrist practices is quite different on the screen…she is less engaged…she is forgetful…treats her with less specialness…It’s less therapeutically useful for her now to be in this screen relationship” (P9, SU-P).

## Discussion

This study provided insight on the experiences of families and youth with mental health and addiction concerns during the COVID-19 pandemic from the perspective of youth, parents, and service providers. The key findings show increased mental health concerns in youth with decreased supports during the pandemic. Parents carried additional burden as they supported their youth in the absence of available and adequate mental health services. Participants from all three groups described inadequate and inequitable mental health care for youth and families during the pandemic related to service access.

One of the findings from our study highlighted that there were increased concerns related to mental health, substance use, technology use, and suicidal risk in youth during the pandemic. This supports the results of other studies showing the negative impact of the pandemic on youth mental health (Cost et al., 2021; Hawke et al., 2020; Jones et al., 2021). The disruption of mental health and addiction services amid higher needs of youth and families during COVID-19 was an important finding in our study. Our findings provide some insights on barriers impacting mental health service access for youth and families. Levesque et al. (2013) conceptualize service access across five dimensions: (1) approachability (information, outreach, and awareness of services); (2) acceptability (professional values and sociocultural factors impacting service use); (3) availability (characteristics of services, facilities, and providers including geographic location, in-person versus online, wait times, and ability to reach service); (4) affordability (costs of services and ability to pay); and (5) appropriateness (fit between services and need, quality of care, and ability to engage). This study identified barriers to access across all five dimensions. All stakeholders described inadequate information on available mental health services, lack of developmentally and culturally responsive services to meet the needs of youth and parents, unavailable public services with even greater gaps for specialized areas, unavailable public sector services leading to reliance on unaffordable private care, and concerns about quality of care for both youth and parents.

A second finding underlined increased stress and burden for parents supporting a youth with mental health and/or addiction-related concerns during COVID-19. Limited treatment and services for youth and families contribute to *caregiver burden*, which has been described as feeling overwhelmed, frustrated, helpless, isolated, and physically and emotionally exhausted (Miller et al., 2017). During the pandemic, caregivers in our study had increased isolation, responsibilities, and financial concerns. Gadermann et al. (2021) examined the impacts of the pandemic on families and found that parents with children under 18 at home had deteriorated mental health due to COVID-19 and worries about their children’s mental health. Participants discussed policies that existed prior to the pandemic such as confidentiality and consent, but these policies created additional challenges for parents trying to access services for youth in the context of the pandemic. Parents also identified more recent COVID-19 policies that excluded them from their youth’s care and in some instances carried safety risks (i.e., not being able to accompany a youth with suicidal risk to emergency). Youth participants described the need for service providers to expand their practice and involve caregivers in mental health services using cultural and racial sensitivity with the goal of providing psychoeducation about mental health. Family psychoeducation is an evidence-based practice that includes information, skill building, and coping strategies, as well as peer and professional support (Lucksted et al., 2012).

There has been ample evidence showing the important role of families in mental health and addiction recovery and the positive impact on mental health outcomes (Lucksted et al., 2012; MHCC, 2015), but they continue to be excluded from youth mental health services (Miller et al., 2017). Hawke et al. (2021b) examined service preferences for caregivers of youth and young adults aged 14–29 and found that caregiver involvement in treatment was identified as a strong priority. The increased mental health needs of youth and burden on caregivers have led to the development of navigation services which offer support to caregivers and ensure youth and families are matched with appropriate mental health and addiction services to meet their needs (Markoulakis et al., 2019, 2020). The service users in this study described the benefits of having a navigator help youth and families identify and access services. Navigators in our study described challenges helping youth and families during the pandemic because mental health services were simply unavailable. According to Williams (2018), families have an important role to care for the well-being and health of its members and they are “living under conditions of siege” as the “state withholds and withdraws support” (p. 82). The author recommends building resistance against the larger system that excludes caregiving families by listening to families, uniting with families, and collectively engaging in community action to redefine practices and policies that include families.

A third finding showed that there were inadequate and inequitable mental health services for youth and their families. Participants described disparities linked to economic resources, racism, discrimination, and disability (i.e., mental illness, addictions, and neurodevelopmental disorders) and these disparities need to be addressed in part through improved access to mental health care (Ashcroft et al., 2021). This study showed that this was not the case for participants in this study, who noted that even though mental health needs increased, access to mental health services decreased. Youth participants reported that their parents were hesitant about getting involved with services and they attributed this to ethnoracial values and experiences of racism and discrimination in the mental health care system. Williams (2009) found that mental health services are often not culturally responsive to the needs of diverse ethnoracial groups, and this impacts service access.

Our results are consistent with the findings of a recent survey that found widening mental health inequities among Canadians during the pandemic. Although this study did not focus specifically on youth, it found declining mental health and coping abilities in Canadians with disproportionate impacts on specific groups and linked this to social determinants of health (Jenkins et al., 2021). The authors emphasize the urgent need to address social determinants and structural barriers and propose syndemics theory which examines “how health and social disparities emerge from the interactions between disease states and the social, environmental, and economic forces that worsen disease outcomes” (Jenkins et al., 2021, p. 10). Our findings show that for youth and families represented in our study, the responses to COVID-19 did not adequately mitigate the mental health impacts of the pandemic as there was little focus on social determinants of health, and there did not appear to be a commitment to and priority placed on reducing disparities and inequities (Jenkins et al., 2021). These findings may provide evidence to lobby for additional mental health services for youth and families with mental health policy makers during the pandemic and beyond.

### Strengths and limitations

A key strength of this study is the inclusion of the perspectives of service providers and service users with both youth and parents included in the service user group. Another strength is the collaborative approach where researchers and community partners were able to co-generate knowledge. Researchers had prolonged engagement with youth coping with mental health concerns and families. We addressed transferability by describing youth, parent, and service provider perspectives using thick description. Researcher and data triangulation enhanced trustworthiness and provided greater depth and breadth of understanding the experiences of youth with mental health concerns and their families in the context of COVID-19.

One limitation of the study is that our sample was small, which may limit the perspectives that could be obtained from each participant group. Most parents who participated in this study were mothers, two thirds identified as white, and most were supporting sons who were not engaged in services. All youth participants identified as female, most identified as racialized, and none of these youth had caregivers involved in their mental health care. More community-based research that explores diverse youth and caregiver perspectives on youth mental health and access to mental health care is important for future studies. Studies have shown rising needs for sex- and gender-specific mental health programs (Craig et al., 2021), as well as services that are culturally responsive and implement anti-racist practices (Fante-Coleman & Jackson-Best, 2020). These are also important areas to explore in future research.

## Conclusion

Youth mental health is a significant public health concern with high rates of mental illness, substance use disorders, and suicide in young people aged 16–24 (Sukhera et al., 2017). Researchers and practitioners have been advocating to make youth mental services a priority in Canada, arguing that there is a need to transform services to address biopsychosocial-spiritual-cultural needs and have a smoother and clearer transition between child and adult mental health care (Malla et al., 2018). Our study showed that the mental health care system did not provide adequate and equitable care to youth and their families during the pandemic. At a time when mental health needs were higher, the mental health care system offered less support and poorer quality of care, and this was amplified for specific youth populations and those needing specialized care. For a more equitable response to the pandemic, we need an accessible and integrated mental health care system that shows a commitment to addressing social determinants and reducing health disparities and inequities in access to mental health services.

## Contributions to knowledge

What does this study add to existing knowledge?

- Our findings show that the responses to the COVID-19 pandemic have greatly impacted youth and families with increased mental health and addiction-related concerns in youth and decreased access to services.
- Caregivers had additional burden during the pandemic as they supported youth with greater needs with limited support from mental health services.
- Mental health services for youth and their families during the pandemic were inadequate and inequitable.
- Navigation services are an important innovation to support access to appropriate mental health services for youth and families.

What are the key implications for public health interventions, practice or policy?

- The findings highlight the need for a syndemic and health equity approach that shows a commitment to reducing disparities in mental health by focusing on social determinants and increasing access to quality mental health care.
- Caregivers and youth need to be engaged in youth mental health policy development and service design.
- An equitable public health response to pandemics includes mental health promotion, prevention, and treatment.

## References

[CR1] Arim R, Findlay L, Kohen D (2020). The impact of the COVID-19 pandemic on Canadian families of children with disabilities.

[CR2] Ashcroft R, Menear M, Silveira J, Dahrouge S, Emode M, Booton J, McKenzie K (2021). Inequities in the delivery of mental health care: A grounded theory study of the policy context of primary care. International Journal for Equity in Health.

[CR3] Bartel SJ, Sherry SB, Stewart SH (2020). Self-isolation: A significant contributor to cannabis use during the COVID-19 pandemic. Substance Abuse.

[CR4] Braun V, Clarke V (2006). Using thematic analysis in psychology. Qualitative Research in Psychology.

[CR5] Castro-Ramirez F, Al-Suwaidi M, Garcia P, Rankin O, Ricard JR, Nock MK (2021). Racism and poverty are barriers to the treatment of youth mental health concerns. Journal of Clinical Child & Adolescent Psychology.

[CR6] Cost, K. T., Crosbie, J., Anagnostou, E., Birken, C. S., Charach, A., Monga, S., ... & Korczak, D. J. (2021). Mostly worse, occasionally better: Impact of COVID-19 pandemic on the mental health of Canadian children and adolescents. *European Child & Adolescent Psychiatry*, 1-14. 10.1007/s00787-021-01744-3 PMC7909377 33638005

[CR7] Craig SL, Eaton AD, Leung VWY (2021). Efficacy of affirmative cognitive behavioural group therapy for sexual and gender minority adolescents and young adults in community settings in Ontario, Canada. BMC Psychology.

[CR8] DiCicco-Bloom B, Crabtree BF (2006). The qualitative research interview. Medical Education.

[CR9] Fante-Coleman T, Jackson-Best F (2020). Barriers and facilitators to accessing mental healthcare in Canada for black youth: A scoping review. Adolescent Research Review.

[CR10] Fegert JM, Vitiello B, Plener PL, Clemens V (2020). Challenges and burden of the Coronavirus 2019 (COVID-19) pandemic for child and adolescent mental health: A narrative review to highlight clinical and research needs in the acute phase and the long return to normality. Child and Adolescent Psychiatry and Mental Health.

[CR11] Gadermann AC, Thomson KC, Richardson CG, Gagné M, McAuliffe C, Hirani S, Jenkins E (2021). Examining the impacts of the COVID-19 pandemic on family mental health in Canada: Findings from a national cross-sectional study. BMJ Open.

[CR12] Hawke, L. D., Barbic, S. P., Voineskos, A., Szatmari, P., Cleverley, K., Hayes, E., ... & Henderson, J. L. (2020). Impacts of COVID-19 on youth mental health, substance use, and well-being: A rapid survey of clinical and community samples: Répercussions de la COVID-19 sur la santé mentale, l’utilisation de substances et le bien-être des adolescents: un sondage rapide d’échantillons cliniques et communautaires. *The Canadian Journal of Psychiatry*, 65(10), 701-709. 10.1177/0706743720940562 PMC7502874 32662303

[CR13] Hawke LD, Hayes E, Darnay K, Henderson J (2021). Mental health among transgender and gender diverse youth: An exploration of effects during the COVID-19 pandemic. Psychology of Sexual Orientation and Gender Diversity.

[CR14] Hawke, L. D., Thabane, L., Wilkins, L., Mathias, S., Iyer, S., & Henderson, J. (2021b). Don’t forget the caregivers! A discrete choice experiment examining caregiver views of integrated youth services. *Patient, 14*, 791–802. 10.1007/s40271-021-00510-6 PMC8046579 33855684

[CR15] Jenkins, E. K., McAuliffe, C., Hirani, S., Richardson, C., Thomson, K. C., McGuinness, L., ... & Gadermann, A. (2021). A portrait of the early and differential mental health impacts of the COVID-19 pandemic in Canada: Findings from the first wave of a nationally representative cross-sectional survey. *Preventive Medicine*, *145*, 106333. 10.1016/j.ypmed.2020.106333 PMC9755654 33509605

[CR16] Jones EA, Mitra AK, Bhuiyan AR (2021). Impact of COVID-19 on mental health in adolescents: A systematic review. International Journal of Environmental Research and Public Health.

[CR17] Jull JE, Davidson L, Dungan R, Nguyen T, Woodward KP, Graham ID (2019). A review and synthesis of frameworks for engagement in health research to identify concepts of knowledge user engagement. BMC Medical Research Methodology.

[CR18] Levesque JF, Harris MF, Russell G (2013). Patient-centred access to health care: Conceptualising access at the interface of health systems and populations. International Journal for Equity in Health.

[CR19] Lincoln YS, Guba EG (1985). Naturalistic inquiry.

[CR20] Lucksted A, McFarlane W, Downing D, Dixon L (2012). Recent developments in family psychoeducation as an evidence-based practice. Journal of Marital and Family Therapy.

[CR21] Malla, A., Shah, J., Iyer, S., Boksa, P., Joober, R., Andersson, N., ... & Fuhrer, R. (2018). Youth mental health should be a top priority for health care in Canada. *The Canadian Journal of Psychiatry*, *63*(4), 216-222. 10.1177/0706743718758968 PMC5894919 29528719

[CR22] Markoulakis R, Weingust S, Foot J, Levitt A (2016). The Family Navigation Project: An innovation in working with families to match mental health services with their youth’s needs. Canadian Journal of Community Mental Health.

[CR23] Markoulakis R, Chan S, Levitt A (2019). Identifying the key features and outcomes of family navigation services for mental health and/or addictions concerns: a Delphi study. BMC Health Services Research.

[CR24] Markoulakis R, Chan S, Levitt A (2020). The needs and service preferences of caregivers of youth with mental health and/or addictions concerns. BMC Psychiatry.

[CR25] Mental Health Commission of Canada (2015). Taking the next step forward: Building a responsive mental health and addictions system for emerging adults.

[CR26] Miller K, Sukhera J, Lynch J, Wardrop N (2017). Voices unheard: Exploring the caregiver experience for caregivers of emerging adults with mental illness. Families in Society.

[CR27] Miller K, Hameed S, Sukhera J (2020). Lost together: Experiences of family physicians with emerging adult mental health. Canadian Family Physician.

[CR28] Patton MQ (2002). Qualitative research and evaluation methods.

[CR29] Piat M, Sabetti J (2009). The development of a recovery-oriented mental health system in Canada: What the experience of commonwealth countries tells us. Canadian Journal of Community Mental Health.

[CR30] Sandelowski M (2010). What’s in a name? Qualitative description revisited. Research in Nursing & Health.

[CR31] Singh, S. P., Paul, M., Ford, T., Kramer, T., Weaver, T., McLaren, S., ... & White, S. (2010). Process, outcome and experience of transition from child to adult mental healthcare: Multi-perspective study. *The British Journal of Psychiatry*, 197(4), 305-312. 10.1192/bjp.bp.109.075135 20884954

[CR32] Statistics Canada. (2020). Canadians who report lower self-perceived mental health during the COVID-19 pandemic more likely to report increased use of cannabis, alcohol, and tobacco. https://www150.statcan.gc.ca/n1/pub/45-28-0001/2020001/article/00008-eng.htm. Published May 7, 2020. Accessed June 25, 2020.

[CR33] Sukhera J, Lynch J, Wardrop N, Miller K (2017). Real-time needs, real-time care: Creating adaptive systems of community-based care for emerging adults. Canadian Journal of Community Mental Health.

[CR34] Williams CC (2009). Increasing access and building equity into mental health services: An examination of the potential for change. Canadian Journal of Community Mental Health.

[CR35] Williams CC (2018). Caregiving under siege: An argument for critical social work practice with families. Canadian Social Work.

[CR36] World Health Organization (WHO). (2021). *Social determinants of health to advance equity*. Retrieved from https://www.who.int/publications/m/item/social-determinants-of-health-to-advance-equity. Accessed 15 Oct 2021.

